# Q&A: Food additive intolerance

**DOI:** 10.1186/1741-7015-9-115

**Published:** 2011-10-20

**Authors:** Margitta Worm

**Affiliations:** 1 Department of Dermatology and Allergy, Allergy Center Charité, CCM, Charité - Universitätsmedizin Berlin, Charitéplatz 1, D-10117 Berlin, Germany

## Q&A: Food additive intolerance

### 1) What is food additive intolerance and can you tell us what the most common symptoms are?

Food additive intolerance is a non-IgE mediated food hypersensitivity. The hypersensitivity is induced by the food additives via a **direct **mast cell activation. Although the exact pathophysiology is unknown, various clinical signs are characteristic of food additive intolerance. These include cutaneous symtoms like redness of the skin, urticaria and angioedema as well as other organ related symptoms, such as dyspnea, hypotension or dizziness.

### 2) What are the differences between intolerance and allergy?

Both, IgE-mediated allergy, but also food additive intolerance are mast cell dependent reactions. The release of mast cell mediators like histamine, leukotrienes and others leads to the onset of the above mentioned clinical symptoms, which cannot be distinguished concerning the underlying mechanisms. In the case of an allergic reaction, crosslinking of membrane bound IgE via an allergen is inducing mast cell degranulation. In food additive intolerance a direct action of the additive on the mast cells is proposed, however the exact mechanisms are not known.

### 3) What are the most common food additives that cause intolerance?

The most common food additives to which patients are intolerant are sulfite, sodium benzoate and food colorings. In addition, histamine is often accused of inducing intolerance reactions, however its exact role as an intolerance reaction as such requires more clarification.

### 4) What is the prevalence of food intolerance?

It is estimated that 0.1 - 1.5% of the population may suffer from food additive tolerance. So far the data suggest that intolerance reactions occur more frequently in older patients rather than in young children Further, it is known that drug intolerance towards acetylsalicylic acid (ASA) occurs more frequently in asthmatic patients. However, whether this is also true in respect to the prevalence of food intolerance in asthmatics remains to be determined.

### 5) Is there an age dependent increase in allergy risk and what are the reasons for this?

The risk of developing an allergy to my knowledge does not solely depend on age but rather depends on the allergen and the specific exposure situation of an individual. The lifetime risk for a food allergy probably does decrease rather than increase over time. However, in the case of food additive intolerance, this decrease of the risk over time might not be true. A possible explanation for this might be a change of the gastrointestinal barrier function. In addition the presence of additional cofactors, which can trigger such reactions (intake of drugs like ACE-inhibitors, physical activity, in-take of alcohol), makes the onset of food additive intolerance in later life more likely.

### 6) Are there any co-morbidities that increase the risk of being intolerant to food additives?

As mentioned above, probably patients with moderate to severe asthma are at a higher risk of being of intolerant to food additives. In addition, it is well known that patients who suffer from mastocytosis, a genetic disease where mast cells occur in increased numbers in the skin and other organs have an increased risk to develop systemic-reactions to food additives

### 7) What are the current diagnostic and management strategies for food additive intolerance?

To date, the optimal management strategies for food additive intolerance include the avoidance of an increased intake of food additives in general, in particular in large amounts. For example, a meal with ripened cheese, wine and a colored dessert should be avoided. If a patient has skin and gastrointestinal symptoms, a prophylactic intake of antihistamines might be useful. Diagnostic procedures involve an elimination diet (3 - 4 weeks) followed by double blind-placebo-controlled-food challenge (DBPCFC) tests. Only if the double blind-placebo-controlled-food challenge is positive the diagnosis can be proven and dietary recommendations be made. Previous data of patients suffering from chronic urticaria has indicated a change in diet can facilitate gastrointestinal barrier recovery, which enables the patient to include certain food items step by step again over time again.

### 8) Are there any difficulties in the diagnosis of food additive intolerance?

The major difficulties in the diagnosis of food additive intolerance are that the history of symptoms made by the patients might not be clear. In such cases, a symptom diary might be helpful. It is important to note that *in vivo *tests, such as the skin prick-test, and *in vitro tests *such as determination of specific IgE cannot be used to make the diagnosis. Moreover, other methods such as the cellular activation test (CAST) measuring histamine release and/or the leukotriene pathway production can not be recommended to confirm the diagnosis. Therefore, research in this area is urgently required. This would help to improve the diagnostic methods of food additive intolerance, identify patients at risk and would support the development of new therapeutic strategies. The lack of knowledge in this field is e.g. related to the fact that food additive intolerance cannot be studied well in vitro as mast cell reactivity is different if studied in vitro versus in vivo.

### 9) What does the future hold for the diagnosis and management of food additive allergies?

I am sure that in the future we will be able to come up with novel diagnostic approaches. These might be based on cellular test systems and/or a metabolomic analysis from patients' serum. In particular, mediators and their receptors from the leukotriene pathway should be considered in more detail as data from previous studies suggest that genetic polymorphisms of leukotriene receptors may be influential, as well as deviations within the leukotriene profile might be relevant. In terms of medical strategies, the exact role of the gastrointestinal barrier will need more clarification. If methods can be developed to increase the barrier function of the gut these might also offer novel approaches for food intolerant patients because less absorption or enhanced local degradation of the food additive in the gut would decrease the systemic burden.

### 10) Where can I find out more?

Soost S, Leynaert B, Almqvist C, Edenharter G, Zuberbier T, Worm M. Risk factors of adverse reactions to food in German adults. Clin Exp Allergy. 2009 Jul;39(7):1036-44. Epub 2009 Mar 3

Roehr CC, Edenharter G, Reimann S, Ehlers I, Worm M, Zuberbier T, Niggemann B. Food allergy and non-allergic food hypersensitivity in children and adolescents. Clin Exp Allergy. 2004 Oct;34(10):1534-41

Zuberbier T, Edenharter G, Worm M, Ehlers I, Reimann S, Hantke T, Roehr CC, Bergmann KE, Niggemann B. Prevalence of adverse reactions to food in Germany - a population study. Allergy. 2004 Mar;59(3):338-45

Fiedler EM, Zuberbier T, Worm M. A combination of wheat flour, ethanol and food additives inducing FDEIA. Allergy. 2002 Nov;57(11):1090-1. No abstract available

Ehlers I, Hipler UC, Zuberbier T, Worm M. Ethanol as a cause of hypersensitivity reactions to alcoholic beverages. Clin Exp Allergy. 2002 Aug;32(8):1231-5

Worm M, Vieth W, Ehlers I, Sterry W, Zuberbier T. Increased leukotriene production by food additives in patients with atopic dermatitis and proven food intolerance. Clin Exp Allergy. 2001 Feb;31(2):265-73.

Worm M, Ehlers I, Sterry W, Zuberbier T. Clinical relevance of food additives in adult patients with atopic dermatitis. Clin Exp Allergy. 2000 Mar;30(3):407-14

Brockow K, Jofer C, Behrendt H, Ring J. Anaphylaxis in patients with mastocytosis: a study on history, clinical features and risk factors in 120 patients. Allergy. 2008 Feb;63(2):226-32

Reese I, Zuberbier T, Bunselmeyer B, Erdmann S, Henzgen M, Fuchs T, Jäger L, Kleine-Tebbe J, Lepp U, Niggemann B, Raithel M, Saloga J, Vieths S, Werfel T. Diagnostic approach for suspected pseudoallergic reaction to food ingredients. J Dtsch Dermatol Ges. 2009 Jan;7(1):70-7. Epub 2008 Nov 24. Review

Buhner S, Reese I, Kuehl F, Lochs H, Zuberbier T. Pseudoallergic reactions in chronic urticaria are associ-ated with altered gastroduodenal permeability. Allergy. 2004 Oct;59(10):1118-23

